# P21 A one-two punch? Investigating the potential of phage–antibiotic combination (PAC) therapy using pleurotin and phage K in *Staphylococcus aureus*

**DOI:** 10.1093/jacamr/dlae136.025

**Published:** 2024-08-23

**Authors:** Michaël D Tadesse, Antonia Sagona, Fabrizio Alberti

**Affiliations:** School of Life Sciences, University of Warwick, Coventry CV4 7AL, UK; School of Life Sciences, University of Warwick, Coventry CV4 7AL, UK; School of Life Sciences, University of Warwick, Coventry CV4 7AL, UK

## Abstract

**Background:**

MDR organisms pose a major healthcare challenge. The current limitations on antibiotic discovery have necessitated the search for novel discovery methods and sources as well as non-antibiotic alternatives. The basidiomycete-derived, secondary metabolite pleurotin and its congeners could be a proponent of the former, as these have been shown to be effective against Gram-positive bacteria, while bacteriophages (phages) could be the ultimate non-antibiotic alternative. A very promising application of bacteriophage therapy is phage–antibiotic combination (PAC) therapy, where (cocktails of) phage and conventional antibiotic are employed against problematic bacterial strains. In this project, the combination of pleurotin and bacteriophages targeting *Staphylococcus aureus* was examined.

**Methods:**

Pleurotin was isolated from the basidiomycete *Hohenbuehelia atrocaerulea* grown in YM broth. Purification and structure characterization was performed using flash chromatography, LC-MS, HPLC and NMR spectroscopy. Purified pleurotin was compared with pleuromutilin and vancomycin, in combination with phage K against *S. aureus* (NCTC 9318) in single agent and PAC time–kill assays. The cytotoxicity of pleurotin was assessed using LDH and live/dead viability assays in human diabetic skin fibroblasts in comparison to pleuromutilin, vancomycin and phage K.

**Results:**

Pleurotin, pleuromutilin and vancomycin both showed expected inhibitory activity against NCTC 9318. Reduced growth was seen in most concentrations. Phage K in combination with both pleurotin and vancomycin showed a decrease in growth rates compared with treatment with phage or antibiotic alone whereas adding phage to pleuromutilin yielded an antagonistic effect. Cytotoxicity of pleurotin in human cells was not significantly different to vancomycin and pleuromutilin.

**Conclusions:**

Results suggest that phage K can lower the working MIC for pleurotin and vancomycin. Subsequent work revealing efficiency against clinically relevant strains and biofilms will be necessary.

